# Maternal perceptions of the quality of Care in the Free Maternal Care Policy in sub-Sahara Africa: a systematic scoping review

**DOI:** 10.1186/s12913-020-05755-9

**Published:** 2020-10-01

**Authors:** Monica Ansu-Mensah, Frederick I. Danquah, Vitalis Bawontuo, Peter Ansu-Mensah, Desmond Kuupiel

**Affiliations:** 1 Department of Public Health, Faculty of Health and Allied Sciences, Catholic University College of Ghana, Fiapre, Sunyani, Ghana; 2 University Clinic, Sunyani Technical University, Sunyani, Ghana; 3 St. John of God College of Health, Duayaw Nkwanta, Ghana; 4 Research for Sustainable Development Consult, Sunyani, Ghana; 5 Department of Secretaryship and Management Studies, Faculty of Business and Management Studies, Sunyani Technical University, Sunyani, Ghana; 6 Department of Public Health Medicine, School of Nursing and Public Health, University of KwaZulu-Natal, 2nd Floor George Campbell Building, Durban, 4001 South Africa

**Keywords:** Free maternal healthcare, Maternal health, Pregnant women, Post-natal mother, Healthcare financing, Free healthcare policy, Perceptions, Quality of care, Sub-Sahara Africa

## Abstract

**Background:**

The world aims to achieve universal health coverage by removing all forms of financial barriers to improve access to healthcare as well as reduce maternal and child deaths by 2030. Although free maternal healthcare has been embraced as a major intervention towards this course in some countries in sub-Saharan Africa (SSA), the perception of the quality of healthcare may influence utilization and maternal health outcomes. We systematically mapped literature and described the evidence on maternal perceptions of the quality of care under the free care financing policies in SSA.

**Methods:**

We employed the Arskey and O’Malley’s framework to guide this scoping review. We searched without date limitations to 19th May 2019 for relevant published articles in PubMed, Google Scholar, Web of Science, Science Direct, and CINAHL using a combination of keywords, Boolean terms, and medical subject headings. We included primary studies that involved pregnant/post-natal mothers, free maternal care policy, quality of care, and was conduct in an SSA country. Two reviewers independently screened the articles at the abstract and full-text screening guided by inclusion and exclusion criteria. All relevant data were extracted and organized into themes and a summary of the results reported narratively. The recent version of the mixed methods appraisal tool was used to assess the methodological quality of the included studies.

**Results:**

Out of 390 studies, 13 were identified to have evidence of free maternal healthcare and client perceived quality of care. All the 13 studies were conducted in 7 different countries. We found three studies each from Ghana and Kenya, two each in Burkina Faso and Nigeria, and a study each from Niger, Sierra Leone, and Tanzania. Of the 13 included studies, eight reported that pregnant women perceived the quality of care under the free maternal healthcare policy to be poor. The following reasons accounted for the poor perception of service quality: long waiting time, ill-attitudes of providers, inadequate supply of essential drugs and lack of potable water, unequal distribution of skilled birth attendants, out-of-pocket payment and weak patient complaint system.

**Conclusion:**

This study suggests few papers exist that looked at maternal perceptions of the quality of care in the free care policy in SSA. Considering the influence mothers perceptions of the quality of care can have on future health service utilisation, further studies at the household, community, and health facility levels are needed to help unearth and address all hidden quality of care challenges and improve maternal health services towards attaining the sustainable development goals on maternal and child health.

## Background

Maternal healthcare is a global health priority. Evidence shows that the launch of the Millennium Development Goals (MDGs) in 1990 in response to the global health challenges had contributed to the reduction of maternal deaths by 1.6% per annum in Sub-Sahara Africa (SSA) [1]. The world targeted to reduce maternal mortality by 90% in the MDG era [2]. To achieve the set target, some SSA countries implemented various interventions including free maternal healthcare financing policies [3]. Free maternal healthcare refers to a health financing policy that enables pregnant women to receive antenatal care (ANC), skilled delivery, and post-natal services from health facilities at no cost to the mothers or their families [4, 5]. In Ghana, the free maternal healthcare policy is integrated into the Nation Health Insurance Scheme (NHIS) which finances the provision of drugs and supplies as well as services including caesarean section, and other family health services at no cost to the mother [6]. Despite this, maternal mortality remains a major challenge to date, especially in SSA. Evidenced shows that 99% of all maternal deaths still occur in low-and-middle-income countries, of which 66% occur in SSA [7, 8]. Hence, maternal health issues have once again been captured as part of the sustainable development goals (SDGs).

In the present SDG era, the world targets to reduce maternal deaths to less than 70 per 100,000 live births by 2030 (SDG 3.1) [9] and free maternal healthcare services remain vital to achieving this goal [10]. Free maternal healthcare is in line with the World Health Organization’s (WHO) call for countries to eliminate financial barriers and improve access to healthcare for all who need it irrespective of where one lives, work, and income level [11, 12]. Free maternal healthcare financing policy can help drive the achievement of the SDG 3.1 which stipulates the reduction of maternal mortality to less than 70 per 100,000 live births by 2030 [12]. Nonetheless, the introduction of free maternal healthcare policy has its own challenges [13, 14] particularly, with regards to the quality of care delivered to clients [13–15] though the goal of the free care policy might not to improve the quality of care, but just to increase the numbers of mothers seeking care at health facilities [16].

Although causes of this worrying situation of maternal mortality in SSA countries are multifaceted, the quality of maternal healthcare services rendered to pregnant women under the various types of free maternal healthcare financing policies may be a contributory factor [17, 18]. Perceived service quality (PSQ) from the client’s point of view is an assessment procedure through which the client makes a comparison to the prior expectations to his or her perception about the quality of service rendered to him or her [19]. Mothers perception of the quality of maternal healthcare is partly linked to future healthcare utilization decisions and overall trust in the health system which may have severe implications on maternal health outcomes [20]. Thus, efforts to maintain the quality of care are highly essential [3, 16, 17]. However, to the best of our knowledge, no study has examined literature and described evidence focusing on maternal perceptions of the quality of maternal healthcare in relation to free maternal healthcare financing policies. According to The Lancet Global Health Commission “changing health needs, growing public expectations, and ambitious new health goals are raising the bar for health systems to produce better health outcomes and greater social value. What is needed are high-quality health systems that optimise healthcare in each given context by consistently delivering care that improves or maintains health, by being valued and trusted by all people, and by responding to changing population needs” [21]. With this in mind, we systematically explored literature aimed at providing evidence on maternal’ perceptions of the quality of care in the free maternal care policy in hoping that the findings will influence policy decisions towards improving maternal healthcare delivery in SSA, identify literature gaps for future research.

## Methods

### Purpose of the scoping study

We explored evidence on the implementation of free maternal healthcare financing policies and pregnant women’s perception of the quality of care using a scoping review methodology and presented evidence reported in SSA. A scoping review was more suitable and assisted the documentation of research gaps by mapping literature on a research question of choice as recommended by the enhanced 2005 Arksey and O’Malley’s framework and the Joanna Briggs Institute guidelines [22–24]. A scoping review study prior to the conceptualization of a primary research question or a systematic review and meta-analysis may also be useful [22]. We followed the Preferred Reporting Items for Systematic Reviews and Meta-Analyses modified for Scoping Reviews (PRISMA-ScR) [22, 24–26] to report the study results.

### Identifying the research questions

The research question for this study was: What evidence exists on maternal perceptions of the quality of care in the free maternal care policy in SSA?

Population, content, and context (PCC) framework was used to determine the eligibility of the scoping review question as shown in Table 1.

**Table 1:** PCC framework for defining the eligibility of the studies for the primary research question

P-Population	Pregnant women and post-natal mothers.
C-Concept	Free maternal healthcare services: refers to any health financing policy that allows women the entitlement to receive maternal services during pregnancy, delivery, and post-natal period from health facilities at no cost to the mother or her family [4, 5].
C-Context	Quality of care: an assessment through which the client makes a comparison to the prior expectations to her perception about the quality of service rendered to him or her [19]. For example; availability and supply of essential drugs for free, good provider-client relationship, short waiting times and patients’ satisfaction.

### Literature search

We searched five electronic databases with no date limitations up to 19th May 2019 including PubMed, Google Scholar, Web of Science, Science Direct, and CINAHL with full text via EBSCOhost using a combination of the following keywords: “free maternal healthcare financing”, “healthcare financing”, “maternal healthcare”, “delivery”, “health service”, “pregnant women”, “client”, “women”, “expectant mothers” and “mothers”, “perception”, “perspectives”, “quality” “quality of care”. Boolean terms (AND/OR) were used to separate the keywords and medical subject headings (MeSH) terms included. Language and study design restrictions were removed. The full electronic search strategy can be viewed in Supplementary file 1. We also searched the reference list of the included articles for eligible studies.

### Eligibility criteria

#### Inclusion criteria

The inclusion criteria for this review were as follows:

- Articles presenting evidence in SSA
- Articles that included pregnant women and/or post-natal mothers
- Articles presenting evidence on free maternal healthcare/financing policy
- Articles reporting evidence on quality of care/perceived quality of care
- Articles reporting evidence on the relationship between free maternal healthcare and perceived quality of care
- Quantitative, qualitative, and mix methods study designs

#### Exclusion criteria

The exclusion criteria for this review were as follows:

- Studies conducted in Africa but not classified among the WHO African Region
- Studies targeting all women in reproductive age who are not pregnant or lactating
- Studies reporting evidence on quality of care from funders perspective or representatives of health financing institutions
- Studies reporting evidence on quality of care from providers or health managers perspectives
- Other types of reviews

### Study selection

Guided by the eligibility criteria, MAM conducted the database search and performed the title screening. Following the electronic databases search, duplicates were removed, and the abstracts and full articles independently screened by MAM and FID. Discrepancies at the abstract screening stage between MAM and FID were resolved by discussion among the review team, and DK independently resolved the discrepancies that arose between MAM and FID at the full-text stage.

### Charting the data

The selected studies were thoroughly read for data extraction of bibliographic details: Author and date, study title, study aim, country of study, study setting, study design, study population, type of maternal healthcare financing policy study findings and relevant study finding. Data extraction was first piloted by two independent reviewers (MAM and DK) using five included articles. Discrepancies were discussed and the data extraction form amended then, MAM extracted data from the remaining eight articles.

### Quality appraisal of study methods

We used the 2018 version of the mixed methods appraisal tool (MMAT) to appraise the methodological quality of all included primary studies. MMAT has two compulsory screening questions and five sets of questions for each of the study designs included (non-randomised control, qualitative, mixed-methods, and quantitative descriptive studies) as shown in supplementary file 2. Each included study was appraised using the appropriate study design section recommended in the MMAT. MAM appraised the studies and was guided by DK. We obtained the percentage score for each study by adding all the items rated divided by seven and multiplied by hundred. A quality percentage score of ≤50%, 51–75%, 76–100%, was interpreted as below quality, average and high quality respectively [27].

## Results

**Fig. 1:**
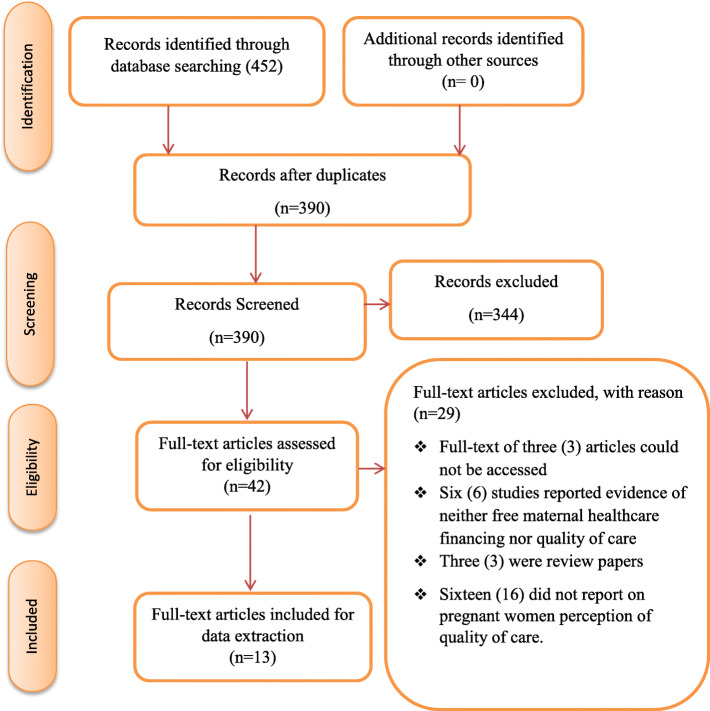
PRISMA 2009 Flow Diagram

Out of the 452 eligible articles obtained from the database search, 62 duplicates were removed. Subsequently, 344 and 29 articles were excluded following the abstract and full article screening stages (Fig. 1). Reasons for exclusion following full article screening were: inability to access the full text of 3 studies [28–30]; one was a protocol [31]; six studies did not present any evidence on free maternal healthcare policy [32–37]; three were review papers [19, 38, 39]; and 15 articles did not report on clients’ perspective of the quality of maternal healthcare [1, 13–15, 20, 40–49]. There was a moderate to a substantial level of agreement between the reviewer’s responses at full article screening stage (Kappa statistic = 0.80, *p* < 0.01).

### Characteristics of included studies

**Fig. 2:**
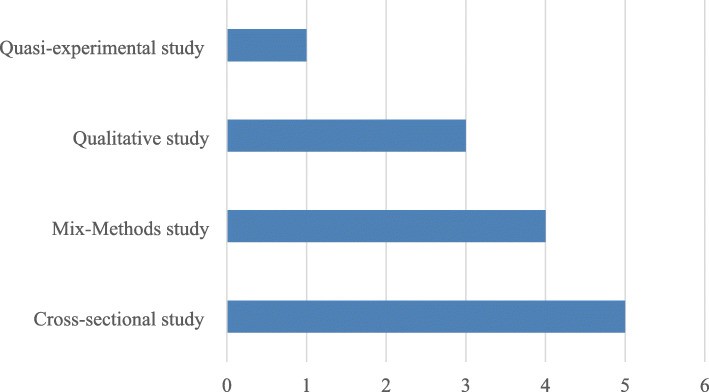
Distribution of the study designs

Of the 13 included articles, 3 (23.1%) each presented evidence from Ghana and Kenya. Two (15.4%) studies each reported evidence from Burkina Faso and Tanzania, and one (7.7%) each reported from Niger, Sierra Leone, and Nigeria. Eight out of the 13 included studies were conducted in health facility-based settings [4, 5, 17, 50–54], two in a household-based based setting [11, 55], two in community-based settings [56, 57], and one was a national survey [58]. The majority (38%) of the included articles were cross-sectional studies [5, 17, 51, 54, 56], whilst the minority (8%) was quasi-experimental study design [52] (Fig. 2). Twelve of the included articles reported evidence on clients’ perspective of the quality of maternal healthcare based on free maternal healthcare financing policies [4, 5, 11, 17, 50–56, 58] and one study based on partially free maternal healthcare financing policy [57].

### Quality of evidence

**Fig. 3:**
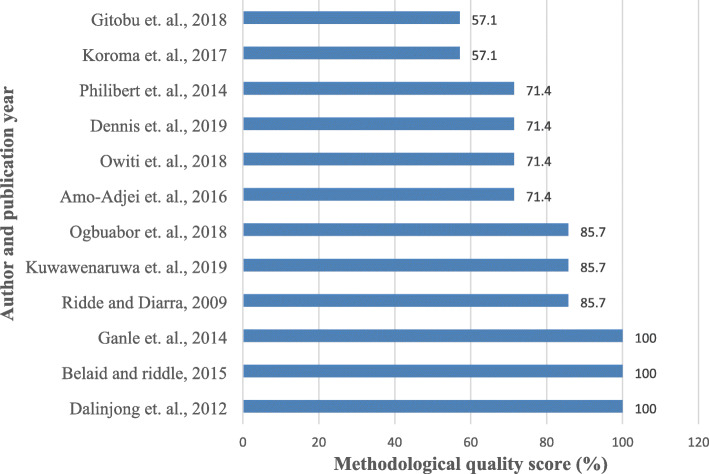
Methodological quality scores of the included articles

Of the 13 included studies, 12 underwent methodological quality assessment using the 2018 MMAT [4, 5, 11, 17, 50–53, 55–58]. All the studies scored between 57.4 and 100%. The majority (33.3%) of the studies scored 71.4% [11, 51, 52, 56] whilst 2 (16.7%) scored the least 57.1% [5, 17]. Figure 3 presents the quality appraisal score per study. One included study did not undergo the quality assessment because it was grey literature [54].

### Study findings

Of the 13 included studies, only five (38.5%) reported evidence of high quality maternal healthcare based on pregnant women’s perspective [5, 11, 52–54]. However, in 8 of the studies, pregnant women were not satisfied with the quality of maternal healthcare rendered under the free maternal care policy [4, 17, 50, 51, 55–58]. Table 2 presents a summary of the study findings from all the 13 included studies.

**Table 2:** Study findings

Author & Date	Study Pop.	Type of healthcare financing policy	Significant study findings	Perception of the quality of care
Amo-Adjei et al., 2016 [56]	Pregnant women	Free maternal healthcare	The quality of care rendered to card bearers of the National Health Insurance Scheme was worse and some form of illegal out-of-pocket payment was found.	Poor
Dalinjong et al., 2012 [4]	Pregnant women	Free maternal healthcare	Clients experienced long waiting times, verbal abuse, and discriminated by providers.	Poor
Koroma et al., 2017 [17]	Pregnant women and health providers	Free maternal healthcare	Inadequate beds, drug supplies, no potable water, and poor reception of providers and low skilled birth attendants.	Poor
Kuwawenaruwa et al., 2019 [55]	Pregnant women	Free maternal healthcare	Reduction of the financial burden for women, poor attitude of providers, ignorance of clients about the policy	Poor
Dennis et al., 2019 [11]	Pregnant women	Free maternal healthcare	Early initiation of ANC visit	Good
Mahamoud, 2017 [54]	Pregnant women	Free maternal healthcare	Available essential drugs, friendly provider-client relationship, clean environment High satisfaction rate	Good
Ogbuabor and Onwujekwe 2018 [58]	Pregnant women, managers, providers	Free maternal healthcare	Distrustful relationships with policymakers and providers, weak patient complaint system (No suggestions box to put it their grievances)	Poor
Owiti et al., 2018 [51]	Pregnant women	Free maternal healthcare	Low utilization of service due to perceived poor quality of care, ill-attitude of provider, fear of being charged for delivery.	Poor
Philibert et al., 2014 [52]	Pregnant women	Free maternal healthcare	High satisfactory rate of service and quality of care, providers give assurance, good nursing care and interaction, and clean environment	Good
Belaid and Ridde, 2015 [57]	Pregnant women, frontline managers, & Providers	Partially free obstetric care	Clients were charged for some drugs meant to be free, providers ill-attitudes and charging of illegal fees coupled with poor quality of care put clients off	Poor
Ridde and Diarra 2009 [53]	Pregnant women, and healthcare providers	Free maternal healthcare	Clients support user-fees abolition	Good
Gitobu et al., 2018 [5]	Pregnant women	free maternal health policy	More than half (54.5%) of the respondents were satisfied with all indicators, but the majority were unsatisfied with privacy, cleanliness and waiting time.	Mixed perception
Ganle et al., 2014 [50]	Pregnant women & health providers	Free maternal healthcare	Poor quality of care due to long lower staff strength, limited and unequal distribution of skilled workers	Poor

### Perceived high quality maternal healthcare

Pregnant women’s perspectives on the quality of care were based on several good services provided to them. A study conducted in Kenya aimed to understand how the implementation of free maternal healthcare policy at the national level resulted in variable effects (coverage of facility-based deliveries) and reported that there was a comparable decrease in discontinuous care across maternal health continuum [11]. Their study also revealed that more women sought maternal healthcare services during the first trimester and subsequently due to their perceived high quality of the services rendered under the free maternal healthcare policy [11]. A study by Mahamoud (2017) in Tanzania discovered that there was high client satisfaction as pregnant women reported that essential drugs and supplies were always available and women experienced good provider-client relationships [54. Gitobu et al. (2018); Philibert et al.; and (2014) Mahamoud (2017), studies respectively in Kenya, Tanzania, and Burkina Faso revealed that pregnant women were satisfied with the cleanliness of the health facilities environment [5, 52, 54]. Women from Tanzania attested to the fact that there existed: good providers-client relationships, assurance of safe delivery and care, and good nursing care [54]. Gitobu et al. (2018); study showed that 54.5% of the pregnant women were satisfied with the quality of maternal health service in Kenyan health facilities under the free maternal healthcare [5]. Their study further evinced that pregnant women appreciated the explanations on the drug administration process; organized and well-structured drug supply; provision of bed net and warm water supply during their stay in the facility [5]. In Burkina Faso, Philibert and colleagues study found that pregnant women were highly satisfied with the service and quality of care [52]. Pregnant women also indicated that providers gave them assurance, good nursing care, and interaction, and the cleanliness of the environment was good [52]. Ridde and Diarra study in Niger aim to describe the importance of the abolition of user-fee for pregnant women and children under five, also reported that pregnant women indicated that the introduction of the free maternal healthcare policy gave them a peace of mind in seeking healthcare services [53].

### Perceived low quality of maternal healthcare

Despite the commendations of the quality of maternal healthcare delivery by pregnant women in some of the included studies, the majority of the included studies revealed low or poor quality of care under the free maternal healthcare policy. In eight of the studies included in this review, pregnant women complained that the quality of services rendered to them under the free maternal healthcare was of low quality owing to several reasons [4, 17, 50, 51, 55–58]. Amo-Adjei et al., and Belaid and Ridde in their respective studies in Ghana and Burkina Faso revealed that out of pocket (OOP) still existed despite the implementation of free maternal healthcare [56, 57]. Amo-Adjei et al. reported that the quality of care rendered to card bearers of the national health insurance scheme (NHIS) including expectant mothers was worse [56]. In Burkina Faso, Belaid and Ridde study revealed that expectant mothers were complaining of fees charged them for some [57] drugs meant to be free and other illegal charges, and poor quality of care. Various studies conducted in Sierra Leone, Kenya, Burkina Faso, and Tanzania revealed that mothers were faced with ill-attitudes of providers [17, 51, 55, 57] just because free maternal insurance is seen as intervention meant for the poor [56] . Not only that women who received free maternal care suffered from verbal abuse [4] and poor reception by providers [17]. Dalinjong et al.; and Ganle et al. in their respective studies in (2013) and (2014) in Ghana, showed there was an assumption that the huge workload of the providers influenced their unfriendly attitudes towards the insured mothers [4, 50]. Insured mothers experienced long waiting times and discrimination by the providers because an immediate payment was not made by them [4, 50].

## Discussion

This scoping review was conducted to explore evidence of free maternal healthcare services and pregnant women’s perspectives on the quality of healthcare delivery in SSA. The results revealed limited [13] studies reporting evidence on the perspectives of pregnant women on the quality of care under the free maternal healthcare policy in SSA. We found evidence from seven countries of which six (85.7%) were in West Africa. The study findings also showed the majority (61.5%) of the included studies reported that pregnant women were not satisfied with the quality of care [4, 17, 50, 51, 55–58] rendered under the free maternal care policy.

**Fig. 4:**
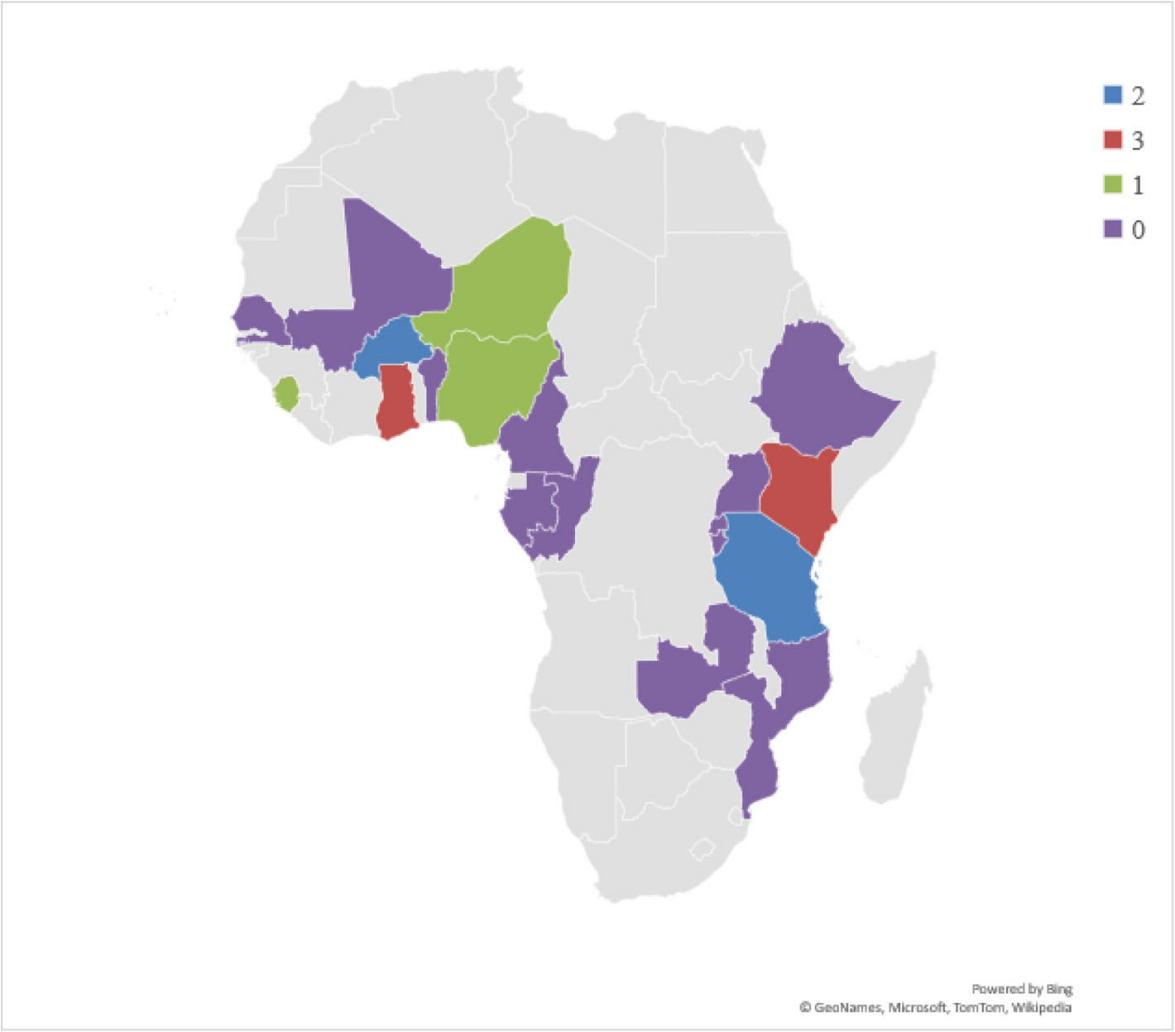
Geographical locations of SSA countries with some forms of free maternal healthcare policies and the number of studies included in this study. (Note: The Authors generated ths map using Microsoft Excel hence, it is freely available to use)

We found only 13 studies that presented information on the perspectives of pregnant women and the quality of care under the free maternal healthcare policy. Although this is the first study to extensively examine the literature on this topic, our finding suggests there is limited evidence. Pregnant women’s perception of the quality of care is a key performance metric of health services hence, it is worth studying since it can influence service utilization [59, 60]. Additionally, as healthcare stakeholders continue to search for initiatives that improve positive pregnancy experiences and outcomes, this study’s results have demonstrated the need for more researches to inform policy decisions and improve maternal healthcare [61]. Evidence shows that some forms of free maternal health services exist in 19 SSA countries [62–65]. But based on this study’s inclusion criteria, we found evidence from only 7 countries representing about 37% of the countries (Fig. 4). Perhaps, our search strategy in our choice of databases and language limitations is the reason or no published study existed on this study’s population, concept, and context in those countries at the time of the literature search for this study. Nonetheless, one recommended intervention to achieve SDG 3 including universal health coverage is the removal of financial barriers to improve access to healthcare to all who require it [9, 27].

This study further found that majority of the included studies reported on perceived poor quality of healthcare under the free maternal health financing policy. This finding calls for concern as this may affect future use of maternal health services at the facilities. Reasons for the perceived poor quality of care include illegal charges by health workers, selling of drugs covered by the free maternal healthcare policy, ill-attitudes of providers, and poor sanitation [17, 50, 51, 55]. Efforts to address these maternal delivery challenges will potentially improve use of maternal healthcare services, and thus improve maternal health outcomes. Whilst delay in the reimbursement of the health facilities may contribute to the perceived poor quality such as an inadequate supply of essential drugs and supplies, and out-of-pocket payment and lack of potable water are essential to explain to clients the basis for any charge or fee to avoid confrontations [66, 67]. Decentralizing maternal health services and resourcing health facilities at the primary healthcare level to enable them render essential services may also help address challenges leading to perceived long waiting times, and lack of privacy during care found by this study [68].

### Implication for practice

Our study findings have several implications for practice. Most of the included studies indicated that pregnant women perceived the quality of care as low. This may encourage some pregnant women to patronize the services of traditional birth attendants or other self-acclaimed midwives residing in their communities. Illegal charges and selling of drugs to clients could also result in the general feeling that free maternal healthcare is not a reality and may cause some disaffection for maternal healthcare providers, hospital/clinic managers, and governments. Illegal charges and/or drugs selling may also discourage some women from enrolling for the service where registration is a prior requirement in some countries. Also, the ill-attitudes of care providers reported by some of the included studies may imply that some pregnant women will find it difficult in expressing their sentiments and grievances to the care providers as well as establishing good relationship with them. Delay in complains and access to health providers also account for complications. Moreover, most women in the SSA do not have formal education and are not able to read or understand the patient charter as well as the free maternal healthcare package [55]. Therefore, women’s privacy may be abused, and flexible appointments could not be made. Again, some studies reviewed, reported a shortage of drugs and inadequate supply of essential drugs and supplies [17, 57]. Meanwhile, the provision of essential drugs and supplies is part of the package of free maternal care. This implies that women’s standard of care was below standard as pregnant women cannot be provided with drug at the required prescription and at the appropriate time. Also, shortage and inadequacy of drugs will result in OOP [56, 57]. To address these implications for practice and ensure free policies meet the goals, we recommend a regular key stakeholder engagement to openly identify and address challenges confronting the free policy. Frequent monitoring and evaluation of the free policy and how it is yielding the need results by the governments through their implementing agencies and the fund managers would also be beneficial. Also, early reimbursement of health facilities is crucial to avoid stock-outs of medical supplies including medicines in health facilities. We further recommend that realistic prices of medical consumables or cost of services need to be paid to health facilities to prevent top-up charges demanded by health facilities. Moreover, we recommend that health facilities or health providers who are found indulging in fraudulent activities inimical to the free maternal healthcare policy should be punished to serve as a deterrent to others.

### Implication for research

Our study indicates limited publications specifically on free maternal healthcare financing and quality of care in SSA. Among the 46 SSA countries in the WHO Africa region, only 7 countries have publication on the topic which indicates a wide gap in the literature. Also, most of the studies were also conducted at the health facility level and few of them being at the household and community-levels. We anticipate that this study will motivate other researchers to conduct more primary studies aimed at investigating further on free maternal healthcare financing and pregnant women’s perception of the quality of care in SSA. We also recommend that further researches should target interviewing the study participants at the household or community-level to elicit other concerns bothering on the quality of maternal healthcare which probably will be hidden at the facility level. Although this study focused on maternal perceptions of the quality of care in free care policy, it is possible other studies exist that focused on the causal impact of free maternal care policies on outcomes, but they do not focus on quality. Hence, we further recommend a follow-up systematic review and meta-analysis to determine the impact of these free maternal care policies on maternal health outcomes in SSA.

### Strengths and limitations

This scoping review is an extensive study to map evidence on free maternal healthcare and the quality of care in SSA. The study established a significant gap in literature relating to free maternal healthcare financing and the quality of care in SSA. The study methodology allows as to include different study designs. Nonetheless, this study has many limitations. We searched few databases for relevant studies; therefore, it is possible other relevant studies that existed in other databases either those we searched were missed. It is also possible other relevant studies on maternal perceptions of quality of care of a free program existed under different terminologies that were not captured by this review electronic search strategy. However, MeSH terms or subject heading and Boolean terms were combined with the keywords appropriately during the electronic search to address this limitation. Additionally, using meta-analysis could provide further information with quantitative studies. Conversely, due to the empirical nature of this scoping review, meta-analysis for quantitative studies was not conducted. Major healthcare stakeholders such as health providers, managers, health insurance or fund managers, and frontline manager perceptions of the quality of care under the free maternal healthcare policy are also essential but this study was limited to only women’s perspective. We recommend future follow-up researches to address these limitations. Despite these limitations, we have provided evidence to guide future researches in SSA.

## Conclusion

This study demonstrated limited evidence in the literature. The expectations of pregnant women such as short waiting time, privacy and confidentiality, elimination of out-of-pocket, provision of potable water, friendly attitude of providers, regular drug supply and non-existence of verbal abuse still remain impediments towards achieving quality maternal healthcare. Governments in SSA countries should join hands with key stakeholders such as healthcare managers, policymakers, and financing agencies to address the various challenges in order to render quality services to women. Moreover, problems like long waiting times could be addressed by using paperless systems and software to limit cumbersome procedures involved in the filling of forms and retrieval of folders. Again, healthcare managers so should build capacities of maternal healthcare providers in order to strengthen the relationship between caregivers and pregnant women. Lastly, financial assistance should also be made available by the government on a regular basis to meet the challenging demands of health facilities.

## Supplementary information

**Additional file 1: Supplementary file 1.** Electronic databases search results for title screening.

**Additional file 2: Supplementary file 2.** Mix Method Quality Appraisal Tool.

## Data Availability

The data supporting the conclusion of this paper are available through the detailed reference list. No original datasets are present since this is a review of the existing literature.

## Abbreviations

ANC: Antenatal care
MDG: Millennium development goal
MMAT: Mixed method quality appraisal tool
NHIS: National Health Insurance Scheme
OOP: Out-of-pocket
PCC: Population, content, and context
PRISMA-ScR: Preferred reporting items for systematic reviews and meta-analyses modified for scoping reviews
PSQ: Perceived service quality
SDG: Sustainable development goal
SSA: Sub-Sahara African
WHO: World Health Organization

## References

[1] Twum P, Qi J, Aurelie KK, Xu L. Effectiveness of a free maternal healthcare programme under the National Health Insurance Scheme on skilled care: evidence from a cross-sectional study in two districts in Ghana. BMJ Open. 2018;8(11). 10.1136/bmjopen-2018-022614 PMC6231580 30413503

[2] Hogan MC, Foreman KJ, Naghavi M, Ahn SY, Wang M, Makela SM (2010). Maternal mortality for 181 countries, 1980-2008: a systematic analysis of progress towards millennium development goal 5. Lancet..

[3] Weimann E, Stuttaford MC. Consumers' perspectives on national health insurance in South Africa: using a mobile health approach. JMIR mHealth and uHealth. 2014;2(4):e49. 10.2196/mhealth.3533 PMC4259968 25351980

[4] Dalinjong PA, Laar AS (2012). The national health insurance scheme: perceptions and experiences of health care providers and clients in two districts of Ghana. Health Econ Rev.

[5] Gitobu CM, Gichangi PB, Mwanda WO. Satisfaction with delivery services offered under the free maternal healthcare policy in Kenyan public health facilities. J Environ Public Health. 2018;2018. 10.1155/2018/4902864 PMC5987322 29951103

[6] Weimann E, Stuttaford MC (2014). Consumers’ perspectives on national health insurance in South Africa: using a mobile health approach. JMIR mHealth uHealth.

[7] World Health Organization. World health statistics 2016: monitoring health for the SDGs sustainable development goals. Geneva: World Health Organization; 2016.

[8] Kuupiel D, Bawontuo V, Drain PK, Gwala N, Mashamba-Thompson TP (2019). Supply chain management and accessibility to point-of-care testing in resource-limited settings : a systematic scoping review.

[9] Gaffney O (2014). Sustainable development goals: improving human and planetary wellbeing. Glob Chang.

[10] Witter S, Adjei S, Armar-klemesu M, Graham W, Witter S, Adjei S (2009). in Ghana.

[11] Dennis ML, Benova L, Abuya T, Quartagno M, Bellows B, Campbell OM. Initiation and continuity of maternal healthcare: examining the role of vouchers and user-fee removal on maternal health service use in Kenya. Health Policy Plan. 2019;34(2):120–31. 10.1093/heapol/czz004 PMC6481282 30843068

[12] Sachs JD (2012). From Millennium Development Goals to Sustainable Development Goals.

[13] Pyone T, Smith H, van den Broek N (2017). Implementation of the free maternity services policy and its implications for health system governance in Kenya. BMJ Glob Heal.

[14] Nimpagaritse M, Bertone MP (2011). The sudden removal of user fees: the perspective of a frontline manager in Burundi. Health Policy Plan.

[15] Witter S, Arhinful DK, Kusi A, Zakariah-Akoto S (2007). The experience of Ghana in implementing a user fee exemption policy to provide free delivery care. Reprod Health Matters.

[16] Chou D (2010). Ending preventable maternal and newborn mortality and stillbirths.

[17] Koroma MM, Kamara SS, Bangura EA, Kamara MA, Lokossou V, Keita N. The quality of free antenatal and delivery services in Northern Sierra Leone. Health Res Policy Syst. 2017;15(1):13–20. 10.1186/s12961-017-0218-4 PMC5516841 28722561

[18] Machira K, Palamuleni M (2018). Women ’ s perspectives on quality of maternal health care services in Malawi.

[19] Meessen B, Hercot D, Noirhomme M, Ridde V, Tibouti A, Tashobya CK (2011). Removing user fees in the health sector: a review of policy processes in six sub-Saharan African countries. Health Policy Plan.

[20] Johnson FA, Frempong-ainguah F, Padmadas SS (2016). Two decades of maternity care fee exemption policies in Ghana : have they benefited the poor ?.

[21] Kruk ME, Gage AD, Arsenault C, Jordan K, Leslie HH, Roder-DeWan S, Adeyi O, Barker P, Daelmans B, Doubova SV, English M. High-quality health systems in the Sustainable Development Goals era: time for a revolution. Lancet Glob Health. 2018;6(11):e1196–252. 10.1016/S2214-109X(18)30386-3 PMC7734391 30196093

[22] Arksey H, O’Malley L (2005). Scoping studies: towards a methodological framework. Int J Soc Res Methodol Theory Pract.

[23] Joanna Briggs Institute. The Joanna Briggs Institute best practice information sheet: Music as an intervention in hospitals. Nurs Health Sci. 2011;13(1):99–102. 10.1111/j.1442-2018.2011.00583.x 21426462

[24] Arksey H, O'Malley L. Scoping studies: towards a methodological framework. Int J Soc Res Methodol. 2005;8(1):19–32.

[25] Tricco AC, Lillie E, Zarin W, O’Brien K, Colquhoun H, Kastner M, Levac D, Ng C, Sharpe JP, Wilson K, Kenny M. A scoping review on the conduct and reporting of scoping reviews. BMC Med Res Methodol. 2016;16(1):15. 10.1186/s12874-016-0116-4 PMC4746911 26857112

[26] Daigneault PM, Jacob S, Ouimet M (2014). Using systematic review methods within a Ph.D. dissertation in political science: challenges and lessons learned from practice. Int J Soc Res Methodol.

[27] Kuupiel D, Tlou B, Bawontuo V, Mashamba-Thompson TP (2019). Accessibility of pregnancy-related point-of-care diagnostic tests for maternal healthcare in rural primary healthcare facilities in northern Ghana: a cross-sectional survey. Heliyon..

[28] Lang E, Mwanri L, Temmerman M (2019). Effects of free maternity service policy in Kenya : an interrupted time series analysis. Lancet Glob Heal.

[29] Yates R (2009). Universal health care and the removal of user fees. Lancet..

[30] Green A (2016). Experts sceptical about Nigeria ’ s free health-care plans Nigeria ’ s government has promised to provide health coverage to millions of citizens. But experts Lancet.

[31] Borghi J, Ramsey K, Kuwawenaruwa A, Baraka J, Patouillard E, Bellows B (2015). Protocol for the evaluation of a free health insurance card scheme for poor pregnant women in Mbeya region in Tanzania: a controlled-before and after study. BMC Health Serv Res.

[32] Agyapong A, Afi JD, Kwateng KO. Examining the effect of perceived service quality of health care delivery in Ghana on behavioural intentions of patients: the mediating role of customer satisfaction. Int J Healthc Manag. 2018;11(4):276–88.

[33] Emelumadu OF, Onyeonoro UU, Ukegbu AU, Ezeama NN, Ifeadike CO, Okezie OK. Perception of quality of maternal healthcare services among women utilising antenatal services in selected primary health facilities in Anambra State, Southeast Nigeria. Niger Med J. 2014;55(2):148. 10.4103/0300-1652.129653 PMC4003719 24791050

[34] Arthur E. Wealth and antenatal care use: implications for maternal health care utilisation in Ghana. Health Econ Rev. 2012;2(1):14. 10.1186/2191-1991-2-14 PMC3484029 22866869

[35] Babalola S, Fatusi A. Determinants of use of maternal health services in Nigeria-looking beyond individual and household factors. BMC Pregnancy Childbirth. 2009;9(1):43. 10.1186/1471-2393-9-43 PMC2754433 19754941

[36] Asundep NN, Carson AP, Turpin CA, Tameru B, Agidi AT, Zhang K (2013). Determinants of access to antenatal care and birth outcomes in Kumasi, Ghana. J Epidemiol Glob Health.

[37] De Allegri M, Tiendrebéogo J, Müller O, Yé M, Jahn A, Ridde V (2015). Understanding home delivery in a context of user fee reduction : a cross-sectional mixed methods study in rural Burkina Faso.

[38] Hatt LE, Makinen M, Madhavan S, Conlon CM. Effects of user fee exemptions on the provision and use of maternal health services: a review of literature. J Health Popul Nutr. 2013;31(4 Suppl 2):S67.24992804

[39] Witter S, Garshong B, Ridde V (2013). An exploratory study of the policy process and early implementation of the free NHIS coverage for pregnant women in Ghana. Int J Equity Health.

[40] Wamalwa EW. Implementation challenges of free maternity services policy in Kenya: the health workers’ perspective. Pan Afr Med J. 2015;22(1). 10.11604/pamj.2015.22.375.6708 PMC4796785 27047616

[41] Witter S, Dieng T, Mbengue D, Moreira I, De Brouwere V (2010). The national free delivery and caesarean policy in Senegal: evaluating process and outcomes. Health Policy Plan.

[42] Okonofua F, Lambo E, Okeibunor J, Agholor K (2011). Advocacy for free maternal and child health care in Nigeria—results and outcomes. Health Policy (New York).

[43] Dalinjong PA, Wang AY, Homer CSE.Dalinjong PA, Wang AY, Homer CSE. The implementation of the free maternal health policy in rural northern Ghana: synthesised results and lessons learnt. BMC Res Notes. 2018;11(1):341. 10.1186/s13104-018-3452-0 PMC5975462 29843780

[44] Mills S, Williams JE, Adjuik M, Hodgson A. Use of Health Professionals for Delivery Following the Availability of Free Obstetric Care in Northern Ghana. Matern Child Health J. 2008;12(4):509–18. 10.1007/s10995-007-0288-y 17955355

[45] Ezugwu EC, Onah H, Iyoke CA, Ezugwu FO, Onah H, Iyoke CA (2015). Obstetric outcome following free maternal care at Enugu State University Teaching Hospital ( ESUTH ), Parklane , Enugu , South-eastern Nigeria Obstetric outcome following free maternal care at Enugu State University.

[46] Pearson L, Gandhi M, Admasu K, Keyes EB. User fees and maternity services in Ethiopia. Int J Gynecol Obstet. 2011;115(3):310–5. 10.1016/j.ijgo.2011.09.007 21982855

[47] Lang E, Mwanri L (2015). Healthcare service providers ’ and facility administrators ’ perspectives of the free maternal healthcare services policy in Malindi District , Kenya : a qualitative study.

[48] Nabyonga-Orem J, Karamagi H, Atuyambe L, Bagenda F, Okuonzi SA, Walker O (2008). Maintaining quality of health services after abolition of user fees: a Uganda case study. BMC Health Serv Res.

[49] El-khoury M, Hatt L, Gandaho T (2012). User fee exemptions and equity in access to caesarean sections : an analysis of patient survey data in Mali.

[50] Ganle JK, Parker M, Fitzpatrick R, Otupiri E (2014). A qualitative study of health system barriers to accessibility and utilization of maternal and newborn healthcare services in Ghana after user-fee abolition.

[51] Owiti A, Oyugi J, Essink D. Utilization of Kenya’s free maternal health services among women living in Kibera slums: A cross-sectional study. Pan Afr Med J. 2018;30. 10.11604/pamj.2018.30.86.15151 PMC6191270 30344870

[52] Philibert A, Ridde V, Bado A, Fournier P (2014). No effect of user fee exemption on perceived quality of delivery care in Burkina Faso : a case-control study.

[53] Ridde V, Diarra A (2009). A process evaluation of user fees abolition for pregnant women and children under five years in two districts in Niger (West Africa). BMC Health Serv Res.

[54] Mahamoud KJ (2017). Assessment of the quality and satisfaction of maternity health care services among post-Natal mothers.

[55] Kuwawenaruwa A, Ramsey K, Binyaruka P, Baraka J, Manzi F, Borghi J. Implementation and effectiveness of free health insurance for the poor pregnant women in Tanzania: A mixed methods evaluation. Soc Sci Med. 2019;225:17–25. 10.1016/j.socscimed.2019.02.005 30784847

[56] Amo-Adjei J, Anku PJ, Amo HF, Effah MO (2016). Perception of quality of health delivery and health insurance subscription in Ghana. BMC Health Serv Res.

[57] Belaid L (2015). Contextual factors as a key to understanding the heterogeneity of effects of a maternal health policy in Burkina Faso ?.

[58] Ogbuabor DC, Onwujekwe OE. The community is just a small circle: citizen participation in the free maternal and child healthcare programme of Enugu State, Nigeria. Glob Health Action. 2018;11(1):1421002. 10.1080/16549716.2017.1421002 PMC5774396 29343213

[59] Stein SM, Day M, Karia R, Hutzler L, Bosco JA (2015). Patients’ perceptions of care are associated with quality of hospital care: a survey of 4605 hospitals. Am J Med Qual.

[60] Gishu T, Weldetsadik AY, Tekleab AM (2019). Patients’ perception of quality of nursing care; a tertiary center experience from Ethiopia. BMC Nurs.

[61] Hincapie AL, Slack M, Malone DC, MacKinnon NJ, Warholak TL (2016). Relationship between patients’ perceptions of care quality and health care errors in 11 countries: a secondary data analysis. Qual Manag Health Care.

[62] Richard F, Antony M, Witter S, Kelley A, Sieleunou I, Kafando Y (2013). Fee exemption for maternal care in sub-Saharan Africa: a review of 11 countries and lessons for the region.

[63] Mckinnon B, Harper S, Kaufman JS, Bergevin Y (2015). Removing user fees for facility-based delivery services : a difference-in-differences evaluation from ten sub-Saharan African countries.

[64] Panel AP, Brief P (2010). Maternal Health : Investing in the Lifeline of Healthy Societies & Economics.

[65] Dugle G, Rutherford S. Coping with the Supply-Side Effects of Free Maternal Healthcare Policies in Seven sub-Saharan African Countries: a Systematic Review. Afr J Reprod Health. 2019;23(1):46–54. 10.29063/ajrh2019/v23i1.5 31034171

[66] Aryeetey GC, Nonvignon J, Amissah C, Buckle G, Aikins M (2016). The effect of the National Health Insurance Scheme (NHIS) on health service delivery in mission facilities in Ghana: a retrospective study. Glob Health.

[67] Aikins M, Agyepong IA (2012). Final Health Insurance Claims.

[68] Awoonor-Williams JK, Tindana P, Dalinjong PA, Nartey H, Akazili J (2016). Does the operations of the National Health Insurance Scheme (NHIS) in Ghana align with the goals of primary health care? Perspectives of key stakeholders in northern Ghana. BMC Int Health Hum Rights.

